# Relational Memory at Short and Long Delays in Individuals With Moderate-Severe Traumatic Brain Injury

**DOI:** 10.3389/fnhum.2020.00270

**Published:** 2020-07-10

**Authors:** Emily L. Morrow, Michael R. Dulas, Neal J. Cohen, Melissa C. Duff

**Affiliations:** ^1^Department of Hearing and Speech Sciences, Vanderbilt University Medical Center, Nashville, TN, United States; ^2^Beckman Institute, The University of Illinois at Urbana-Champaign, Urbana, IL, United States; ^3^Interdisciplinary Health Sciences Institutes, The University of Illinois at Urbana-Champaign, Urbana, IL, United States

**Keywords:** traumatic brain injury, relational memory, hippocampus, rehabilitation, assessment

## Abstract

Memory deficits are a common and frequently-cited consequence of moderate-severe traumatic brain injury (TBI). However, we know less about how TBI influences relational memory, which allows the binding of the arbitrary elements of experience and the flexible use and recombination of relational representations in novel situations. Relational memory is of special interest for individuals with TBI, given the vulnerability of the hippocampus to injury mechanisms, as well as a growing body of literature establishing the role of relational memory in flexible and goal-directed behavior. In this study, participants with and without a history of moderate-severe TBI completed a continuous relational memory task for face-scene pairings. Participants with TBI exhibited a disruption in relational memory not only when tested after a delay, but also when tested with no experimenter-imposed delay after stimulus presentation. Further, canonical assessments of working and episodic memory did not correspond with performance on the face-scene task, suggesting that this task may tap into relational memory differently and with greater sensitivity than standardized memory assessments. These results highlight the need for rigorous assessment of relational memory in TBI, which is likely to detect deficits that have specific consequences for community reintegration and long-term functional outcomes.

## Introduction

Memory deficits are a common consequence of moderate-severe traumatic brain injury (TBI) and are among the most frequently-identified targets for intervention following injury (Wilson, 1998; Murray et al., 2001; Vakil, 2005; Cicerone et al., 2011). Deficits in long-term declarative memory (i.e., the encoding, consolidation, and retrieval of information about facts, world knowledge, and autobiographical experiences) are particularly well-documented in TBI (Bigler et al., 1996; Palacios et al., 2013; Rabinowitz and Levin, 2014; Irimia and Van Horn, 2015). Semantic and episodic memory ability have been linked to the hippocampus and surrounding medial temporal lobe structures, which have a well-established and critical role in the formation and retrieval of declarative memories (Squire, 1992; Cohen and Eichenbaum, 1993; Eichenbaum and Cohen, 2001; Duff et al., 2020). In TBI, the hippocampus and medial temporal lobes are highly vulnerable to injury mechanisms. For example, several frequently occurring pathophysiological consequences of TBI (e.g., hypoxia, seizure activity) disproportionately affect the structure and function of the hippocampus, making hippocampal damage one of the most likely consequences of injury (Tate and Bigler, 2000; Vespa et al., 2010; Atkins, 2011; Palacios et al., 2013; Sharp et al., 2014; Irimia and Van Horn, 2015). These hippocampal-dependent declarative memory deficits are of high functional significance, as they can interfere with rehabilitation efforts and community reintegration and independence following TBI (Skidmore, 2015).

Advances in cognitive neuroscience research in recent decades have placed special emphasis on the relational nature of declarative memory. This research has demonstrated that hippocampal damage results primarily in an impairment in relational memory (Cohen and Eichenbaum, 1993; Eichenbaum and Cohen, 2001; Monti et al., 2014; Rubin et al., 2017). Relational memory theory highlights two key roles of the hippocampus in relational memory: (1) the *binding* of arbitrary relations between the elements of experience into durable representations; and (2) the *flexible* expression of these representations in novel settings, i.e., in different contexts from encoding (Eichenbaum and Cohen, 2001; Rubin et al., 2017; Rigon et al., 2020). For example, relational memory allows for the binding of names to new acquaintances (e.g., the arbitrary binding of a therapist’s name to the person) or the meeting of temporal goals (e.g., using representations from past experiences to recall the time of next therapy appointment and arrive before the session begins).

Advances in memory research have also expanded the reach of hippocampal relational memory beyond its established role in long-term memory. A growing body of work from patients with focal hippocampal damage reveals deficits in relational memory processes when there are minimal delays, and even when there are no experimenter-imposed delays at all (i.e., all information needed to complete a task is immediately available and/or on the screen at the same time; Hannula et al., 2006; Olson et al., 2006; Barense et al., 2007; Warren et al., 2010). Converging evidence from fMRI indicates activation of the hippocampus for relational learning over similarly short lags, on the timescale of short term or working memory (Mitchell et al., 2000; Ranganath and D’Esposito, 2001).

In one study of hippocampal relational memory over short and long lags, Hannula et al. (2006) compared the performance of individuals with bilateral hippocampal damage and severe declarative memory impairment (hippocampal amnesia) to healthy comparison participants on a face-scene pairing task. Study and test trials were intermixed continuously so that participants were tested on face-scene pairings they had just seen (a lag of one and a delay of only a few seconds), or face-scene pairings that were from nine trials earlier. Critically, on test trials, participants chose the matching face from an array of three equally familiar faces. Thus, item memory was insufficient for a correct response; participants had to call upon the previously-studied arbitrary relational binding of face and scene (Hannula et al., 2006). As expected, the participants with amnesia were significantly impaired at the long lag. However, in striking contrast to earlier studies of intact working memory in amnesia (i.e., hippocampal amnesia does not impair performance on working memory tasks; Baddeley and Warrington, 1970), participants with amnesia were also impaired relative to healthy participants on memory for face-scene relations at the short lag condition, a delay of just a couple of seconds. These findings indicate that the hippocampus may play a role in the processing of relations irrespective of the timescale. Thus, the authors suggested that the role of the hippocampus in memory may have less to do with timescale (the distinction between long-term and short-term memory) and more to do with the distinction between relational memory (e.g., face-scene pairings) and memory for single items (e.g., single faces or digits; Hannula et al., 2006).

Building on this evidence of hippocampal involvement in relational memory representations across timescales, further work has revealed the role of the hippocampus in other cognitive domains. In particular, the hippocampus is critical for the flexible use of relational memory representations, across long and short lags. This flexibility allows for the dynamic, adaptive use of memory to underpin flexible cognition and broader goal-directed behavior (Rubin et al., 2014). For example, relational memory may be critical to learning new compensatory strategies in therapy and flexibly generalizing those strategies to new situations, or to adjusting behavior in response to consequences (Rigon et al., 2020).

Given that impairments in flexible cognition and goal-directed behavior are well-documented in TBI and are often cited as barriers to community reintegration (Ylvisaker et al., 2005), characterization of hippocampal-dependent relational memory in TBI warrants further attention. Such work would result in better alignment between the literature on memory deficits in TBI, which has focused on the more traditional views and assessments of declarative memory, and the cognitive neuroscience of memory literature, which has emphasized the development of new experimental measures to capture relational memory performance across populations where hippocampal pathology is present (e.g., amnesia, aging, schizophrenia, autism; Hannula and Duff, 2017). For example, many experimental tasks and classic neuropsychological tests of memory (e.g., California Verbal Learning Test; Auditory Verbal Learning Test) have focused on the traditional role of declarative memory in encoding and subsequent retrieval, when performance is assessed *via* recall of verbatim word lists or producing exact replication of figures. The use of tasks that directly test *relational* binding may permit the detection of relational memory deficits following TBI that are not routinely captured with currently-available standardized assessments of memory (Rigon et al., 2020).

To date, there have only been a couple of studies that have examined relational memory in TBI. Rigon et al. (2020) examined relational memory in TBI using a spatial reconstruction task. A growing body of work has linked performance on spatial reconstruction tasks to hippocampal integrity and memory performance (e.g., Clark et al., 2017; Horecka et al., 2018). In the study by Rigon et al. (2020), individuals with chronic, moderate-severe TBI showed a spatial reconstruction impairment relative to healthy peers. Particularly interesting was the finding, from an exploratory analysis, that although spatial reconstruction performance was significantly correlated with scores on the California Verbal Learning Test, more participants with TBI exhibited disruption on the spatial relational memory task than the neuropsychological measure, raising the possibility that current standardized neuropsychological assessments of memory are not sensitive to the full range of relational memory abilities (Rigon et al., 2020). In another study, Monti et al. (2013) assessed relational memory in individuals with a recent or distant history of mild TBI using a non-continuous version of the relational face-scene paradigm (Hannula et al., 2006). Interestingly, middle-aged adults with a mild TBI in the remote past exhibited impaired relational memory when compared to peers without a history of mild TBI. Neuroimaging revealed that these individuals also had smaller hippocampi bilaterally and decreased neural activity during retrieval. These results suggested that even a remote history of mild TBI may produce relational memory deficits and that these deficits might be captured decades later with experimental tasks of relational memory (Monti et al., 2013). Taken together, findings from these studies suggest that relational memory tasks may be more sensitive to post-injury hippocampal relational memory deficits than standardized assessments. However, there is a need for more investigation of continuous relational memory performance, across timescales, following moderate-severe TBI.

The current study is part of a broader ongoing effort to characterize relational memory in individuals with TBI, to develop tasks with sufficient specificity and sensitivity to capture a range of relational memory performances, and to determine the relationship between long-term behavioral outcomes and relational memory deficits (Monti et al., 2013; Rigon et al., 2020). As the first step in this line of work, our primary aim in the current study was to assess relational memory performance at short and long lags in moderate-severe TBI relative to a healthy comparison group using the continuous relational face-scene paradigm in Hannula et al. (2006). We predicted that:

1. Individuals with moderate-severe TBI would exhibit disrupted performance relative to healthy comparison participants on the face-scene task, at both short and long lags.
2. Consistent with the findings of Hannula et al. (2006), we predicted a larger deficit for the TBI group at a long lag relative to the short.
3. Given the heterogeneity of deficit profiles in individuals with TBI, we expected considerable variability in performance at both lags within the TBI group.

An exploratory aim of this study was to determine the relation between performance on the experimental relational memory task and performance on traditional neuropsychological measures of declarative and working memory. As this aspect of the study was only exploratory, we did not make specific predictions about the correspondence between the relational memory task and neuropsychological measures in this population. However, given the task’s established sensitivity to hippocampal-dependent relational memory functioning, to the extent that TBI compromises hippocampal function, this task would be expected to be particularly revealing of impairment in this population.

## Materials and Methods

### Participants

Participants were 41 individuals with moderate-severe TBI (22 females, 19 males) and 41 healthy comparison participants (29 females, 12 males). Six participants with TBI and one comparison participant were left-handed. All participants were between the ages of 18 and 55. Healthy comparison participants were recruited from Nashville and the surrounding areas and had no history of neurological or cognitive disability. The mean age for the participants with TBI and the healthy comparison participants were 37.1 (*SD* = 9.4) and 33.6 (*SD* = 9.7) years, respectively, and did not differ statistically (*t*_(79.94)_ = 1.649, *p* = 0.103). The mean level of education in years for the participants with TBI and the healthy comparison participants were 14.9 (*SD* = 2.1) and 15.7 (*SD* = 1.9), respectively, and did not differ statistically (*t*_(79.21)_ = 1.617, *p* = 0.110).

Participants with TBI were recruited through the Vanderbilt Brain Injury Patient Registry. All participants with TBI were in the chronic phase of injury (>6 months post-injury) and sustained their injuries in adulthood (i.e., after age 18). Thus, participants’ neuropsychological profiles were in the chronic and stable phase (Salmond et al., 2006). Average time since injury was 68.3 months (*SD* = 85.2). Participants with TBI did not have a history of neurological or cognitive disabilities before the qualifying brain injury. TBI severity was determined using the Mayo Classification System (Malec et al., 2007). Participants were classified as having sustained a moderate-severe TBI if at least one of the following criteria was met: (1) Glasgow Coma Scale (GCS) <13 within 24 h of acute care admission (i.e., moderate or severe injury according to the GCS); (2) positive neuroimaging findings (acute CT findings, or lesions visible on a chronic MRI); (3) loss of consciousness (LOC) >30 min; or (4) post-traumatic amnesia (PTA) >24 h. Injury-related information was collected from available medical records and a semi-structured interview with participants.

GCS was available for 32 participants (Median = 7, ranging from 3 to 15); loss of consciousness (LOC) information was available for 36 participants; PTA information was available for 38 participants; acute imaging information was available for 36 participants (33 with positive findings). Causes of injury were motor vehicle accidents (16), falls (7), motorcycle or snowmobile accidents (5), being hit by a car as a pedestrian (5), non-motorized vehicle accidents (4), assault (3), or being hit by a moving object (1). See Table 1 for demographic and injury information for participants with TBI.

**Table 1:** Demographic and injury information for participants with traumatic brain injury (TBI).

ID	Age	Edu	Emp	Etiology	TSO	LOC	Neuroimaging	GCS	PTA
5002	41–45	16	No	Non-motor	217	>30 min	Intracranial hemorrhage	3	>24 h
5003	26–30	16	Yes	Ped vs. auto	15	N/A	SDH	11	>24 h
5005	31–35	16	Yes	MVA	23	>30 min	SAH, IVH	14	>24 h
5006	51–55	12	Yes	MCC	407	>30 min	Intracranial hematoma	N/A	>24 h
5010	31–35	16	Yes	Ped vs. auto	11	N/A	SAH; intracranial hemorrhage	6	>24 h
5011	41–45	12	No	Fall	48	>30 min	SAH; frontotemporal contusion; epidural hematoma	15	>24 h
5013	31–35	18	No	Ped vs. auto	7	No	SAH	15	<24 h
5014	46–50	16	Yes	MVA	180	>30 min	N/A	N/A	>24 h
5016	21–25	16	Yes	MVA	13	>30 min	SAH; SDH	13	>24 h
5017	31–35	16	Yes	Ped vs. auto	163	>30 min	N/A	4	>24 h
5018	36–40	18	Yes	MVA	59	No	SAH	3	>24 h
5019	41–45	16	No	Ped vs. auto	24	>30 min	SAH; SDH	6	>24 h
5020	46–50	16	Yes	MCC	60	>30 min	Negative	N/A	>24 h
5021	36–40	18	No	MVA	25	>30 min	Epidural hematoma; SAH	3	>24 h
5027	26–30	16	No	Fall	10	>30 min	SAH	9	N/A
5028	16–20	12	Yes	MVA	18	>30 min	SAH	6	>24 h
5031	51–55	14	Self	Struck by object	7	No	SDH; SAH; IPH; skull fracture	13	N/A
5034	31–35	16	No	MVA	31	>30 min	SAH	3	>24 h
5037	36–40	12	Yes	MVA	37	<30 min	Diffuse intracranial swelling	3	>24 h
5038	36–40	16	Yes	Fall	18	>30 min	SDH; multifocal hemorrhages; post-traumatic hemorrhagic contusions	N/A	>24 h
5039	36–40	12	Self	MVA	57	>30 min	IVH; SAH	3	>24 h
5040	41–45	12	Yes	MVA	69	>30 min	SDH; SAH	3	>24 h
5041	26–30	16	Yes	MVA	53	No	Negative	10	>24 h
5044	21–25	16	Yes	Non-motor	75	No	SDH; skull fracture	15	>24 h
5046	46–50	18	Yes	Non-motor	46	>30 min	SAH	14	<24 h
5047	26–30	16	Yes	Assault	16	<30 min	SDH	15	<24 h
5048	46–50	16	Yes	MVA	336	>30 min	N/A	N/A	>24 h
5050	31–35	18	Yes	Fall	16	>30 min	SAH	N/A	<24 h
5051	46–50	16	Yes	MVA	7	<30 min	SAH	14	<24 h
5053	46–50	16	Yes	MCC	17	>30 min	IPH, SD hemorrhage	5	>24 h
5056	21–25	12	Yes	Non-motor	30	>30 min	Hemorrhagic shear injury	11	>24 h
5057	21–25	12	Yes	MVA	18	No	SDH	N/A	No
5058	31–35	12	Yes	MCC	17	<30 min	SAH; SDH	8	>24 h
5059	31–35	16	No	MCC	99	N/A	Extra-axial hemorrhage	14	<24 h
5060	36–40	12	No	MVA	115	>30 min	Negative	3	>24 h
5068	21–25	16	Yes	Fall	39	<30 min	N/A	N/A	>24 h
5069	26–30	12	No	Assault	115	<30 min	N/A	N/A	>24 h
5070	46–50	16	Yes	Fall	55	<30 min	SAH	15	>24 h
5074	31–35	12	Yes*	Assault	74	N/A	Parenchymal hemorrhage	4	<24 h
5075	51–55	16	No	Fall	86	>30 min	SAH; SDH; hemorrhagic contusion	3	No
5076	41–45	12	No	MVA	86	N/A	SAH	3	N/A

ID = participant ID number. Age is presented in ranges of 5 years to protect participant identity. Education (edu) reflects years of highest degree obtained. For employment status (Emp), Yes = employed or full-time student, No, unemployed; Self, self-employed. Participant listed as “Yes*” was employed at the time of testing but became unemployed within 1 week of testing. MVA, motor vehicle accident. MCC includes both motorcycle and snowmobile accidents. Non-motor, non-motorized vehicle accident. Ped vs. auto = participant was hit by car while walking or running. Time since onset (TSO) is presented in months. Loss of consciousness (LOC) is presented in minutes (min). SDH, subdural hematoma; SAH, subarachnoid hemorrhage; IPH, intraparenchymal hemorrhage; IVH, intraventricular hemorrhage. Glasgow Coma Scale (GCS) is total score within first 24 hours of acute care admission. PTA, post-traumatic amnesia; h, hours. When a cell reads N/A, that information was not available for the given participant.

We should note that this sample of participants with TBI, as a group, demonstrated better functional outcomes (in terms of employment outcome) than is typically reported in the literature (Gormley et al., 2019). For example, of our 41 participants with TBI, 22 were gainfully employed outside the home, and five were full-time students. Only 12 were unemployed, and two were working from home (e.g., self-employed doing odd jobs, selling clothes online). Our sample’s average educational attainment of 14.9 years was also above the average range for individuals who sustain a TBI (Gauthier et al., 2018).

### Face-Scene Relational Memory Task (FSRT)

#### Stimuli and Design

**Figure 1:**
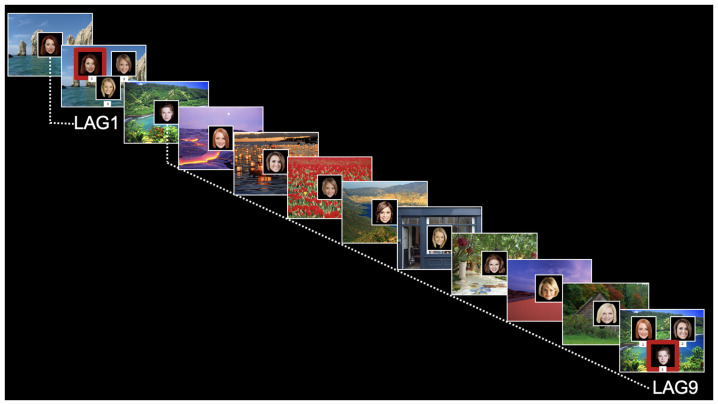
Relational memory task for face-scene pairings. Test trials are marked as Lag1 (short delay) or Lag9 (long delay). Red boxes on test trials are for illustrative purposes only (were not displayed to participants).

The face-scene relational memory task (FSRT) stimuli and design closely followed Hannula et al. (2006). Participants studied two blocks of 36 face-scene pairs (one block containing all male faces and one block containing all female faces), interspersed with 16 test trials (see Figure 1). Test trials included a previously viewed scene superimposed with three previously viewed (and equally familiar) faces. On test trials, participants were asked to identify which face was previously studied with (and therefore matched) the scene presented. Thus, memory for individual items (individual faces) would be insufficient for successful performance. Instead, a correct response would require relational memory for the previously-studied face-scene pairing.

Test trials were placed systematically throughout each block to assess relational memory for face-scene pairings at short lag (Lag1, for the face-scene pairing presented immediately before trial) and long lag (Lag9, for the face-scene pairing presented nine displays previously). Four Lag1 trials and four Lag9 trials were included in each block (for a total of eight trials in each condition). Also, each block contained four “re-pair” test trials, in which none of the faces matched the scene. Note that data from the re-pair displays are not presented here or included in the analysis, as those displays were included in the experiment only for a control condition for a separate eye-tracking version of this paradigm. For both study and test trials, the scene was first shown by itself for 2 s (scene preview). On study trials, the face then appeared superimposed in the center of the scene for 3 s. On test trials, the three faces were presented for 5 s and labeled with the keystroke response. Faces were 300 × 300 pixel images; scenes filled the entire display screen (1,280 × 1,024 pixels). The matching face for test trials appeared equally often in each spatial position (left, right, and bottom).

#### Procedures

Data collection for the FSRT was conducted as part of an initial visit for the Vanderbilt Brain Injury Patient Registry, during which participants complete consent forms and an intake interview. After obtaining informed consent the experimenter explained the task *via* both written and verbal instructions, asking that participants memorize each “study” face-scene pair in anticipation of the presentation of occasional “test” displays. Participants were instructed to respond to “test” displays by pressing the corresponding number key for the face that was previously presented with the test scene. They were encouraged to select a response on every trial, even if they did not believe that any of the faces were studied with that scene (e.g., for “re-pair” trials). Encouraging participants to select a response for every trial, in addition to accounting for re-pair displays, allowed for assessment of response accuracy whereas a failure to respond could occur for multiple reasons (e.g., participants may be unsure but still remember an item and be able to provide an accurate response). “Study” and “test” displays were labeled at the bottom of the screen (i.e., test displays were labeled with “Which is the correct face?”). Before initiation of the FSRT, the participants completed as many practice trials as necessary to ensure that they understood the task and were able to identify the intended face from a three-face display with a key response. Each participant was then tested individually, with a short (few minutes) break between blocks if needed. In total, the FSRT (including training and both experimental blocks) took approximately 30–35 min to administer.

### Neuropsychological Testing

We administered the Working and Episodic Memory subtests from the Cognition Battery of the NIH Toolbox as standardized neuropsychological assessments of memory (Heaton et al., 2014). We chose the NIH Toolbox as a comparison point, as it is widely used and recommended for use in TBI research (e.g., NIH Common Data Elements). Participants completed the Cognition Battery on an iPad during a separate session from the FSRT; the average time between the two sessions was 3.7 weeks. The NIH Toolbox was either administered as the only neuropsychological measure during a given session or as the first measure if other neuropsychological assessments were administered. The Cognition Battery provides individual scores for each construct, as well as composite scores. For standardized neuropsychological memory measures, we utilized age-corrected standard scores from the Working Memory and Episodic Memory subtests. Each subtest took approximately 10–15 min to administer.

The Working Memory (List Sorting) subtest requires immediate recall and manipulation of visually and orally presented information. Participants see images of different foods and animals with accompanying instructions asking the participant to say the items back in size order from smallest to largest, first within a single category (e.g., foods only) and then on two categories (e.g., foods, then animals). The task is scored by summing the total number of correct items, which can range from 0–26, and then converting that score to a nationally normed standard score based on the participant’s age (National Institutes of Health, 2016).

The Episodic Memory (Picture Sequence Memory) subtest involves recalling series of illustrated activities, which increase in length as the subtest continues. Participants must recall the sequence of activities over two learning trials. Sequences vary in length from 6–18 pictures, depending on the participant’s age, and participants receive credit for each adjacent pair of pictures they place correctly. The number of correct adjacent pairs is converted to a theta score, then a nationally normed standard score based on the participant’s age (National Institutes of Health, 2016).

### Statistical Analysis

The dependent variable for the FSRT was the proportion of correct responses (correct identification of matching face/total items) for each participant at Lag1 (immediate test) and Lag9 (delayed test). As the response window was timed for each item, we removed items to which a participant did not respond when calculating the proportion correct. Given ceiling level performance for many participants and concerns about violations of normality, we performed nonparametric statistical tests. To assess group differences at each lag, we conducted a one-sided Mann–Whitney *U* (or Wilcoxon rank-sum) test to compare the ordinal distribution of data points within each group.

We conducted *ad hoc* analyses of group differences in response time at both lags. For these analyses, we used an unequal variances *t*-test, as response times are on a continuous distribution. We only included response time for correct trials (i.e., the trials included in the accuracy proportions). We assessed the relationship between response time and trial accuracy for all participants, using Spearman’s rho to account for violations of normality due to the ceiling performance of many participants on the FSRT.

We conducted exploratory analyses to assess the correlation between performance at Lag1 of the FSRT and scores on the NIH Toolbox Working Memory subtest, as well as the correlation between performance at Lag9 of the FSRT and the NIH Toolbox Episodic Memory subtest, to characterize the relationship between the FSRT and standardized neuropsychological measures. Age-adjusted standard scores from the Episodic and Working Memory subtests were used in the correlation analyses. To account for violations of normality due to ceiling performance, we used Spearman’s rho to assess relations between ordinal rankings of performance on the relational memory and neuropsychological assessments. We conducted exploratory analyses of relationships in performance between the FSRT and the NIH Toolbox, using 1.5 standard deviations below mean performance as a cutoff to assess how deficits would be identified between the two assessments.

All statistical tests were conducted in R, with an alpha of 0.05.

## Results

### Face-Scene Relational Memory Task (FSRT)

Before calculating each participant’s proportion correct, we removed items for which a participant did not respond during the timed response window. For each group, there were 328 possible response items for a given lag (eight items for each of 41 participants). At Lag1, we removed from analysis seven items (2.13% of total items) to which participants with TBI did not respond during the given window. Comparison participants responded to all items at Lag1. At Lag9, we removed 28 items from the analysis (8.54% of total items) for participants with TBI and four items (1.22% of total items) for comparison participants.

Mean performance at Lag9 for participants with TBI and the comparison group was 0.697 and 0.821, respectively. Relative to the comparison group, participants with TBI performed significantly worse on memory for face-scene relations at the long delay (Lag9), *U =* 1,094.5, *p* = 0.008.

**Figure 2:**
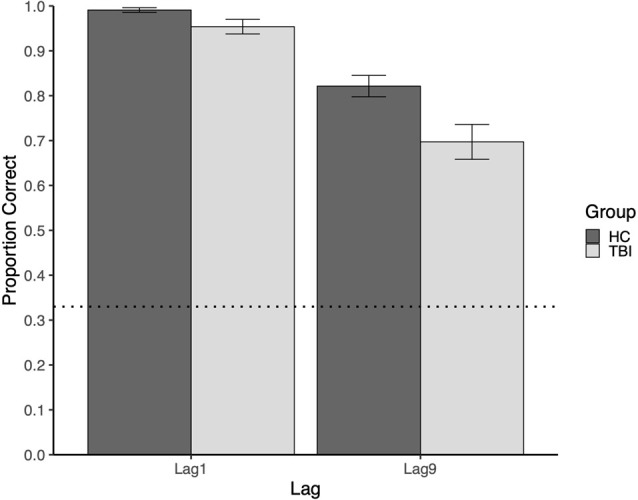
The plot of mean group performance on the Face-Scene Relational Memory Task(FSRT). Error bars represent standard error. The dotted line represents performance at the chance.

Mean performance at Lag1 for participants with TBI and the comparison group was 0.954 and 0.991, respectively. Although there were no intervening items between study and test trials at Lag1, participants with TBI had poorer memory for face-scene pairs than comparison participants, *U* = 971, *p* = 0.025 (see Figure 2). At Lag1, nine participants with TBI had off-ceiling performance, whereas only three comparison participants were off-ceiling^1^. Further, although participants with TBI showed significant disruptions in both lags, this disruption was larger for the long lag (see Figure 2).

**Figure 3:**
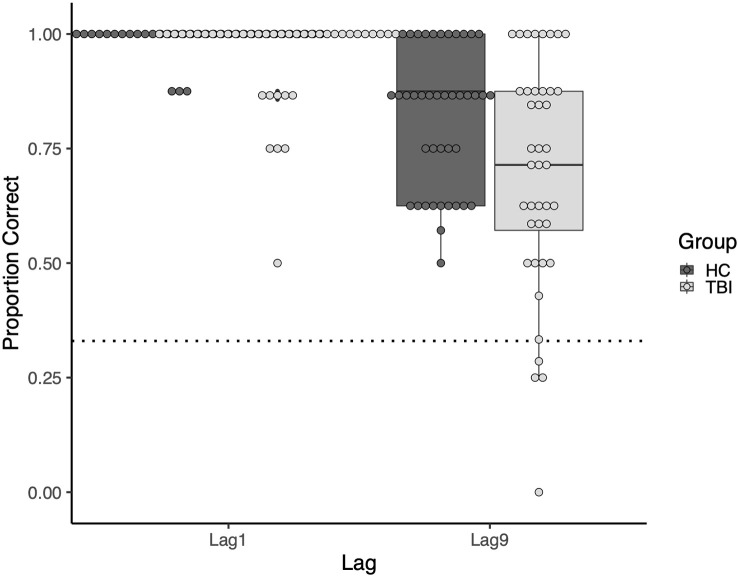
Boxplot of the group and individual performance. Points represent individual participants. The dotted line represents performance at the chance.

At both lags, there was more variability in task performance in the TBI group than in the comparison group. At Lag1, the standard deviation was 0.104 in the TBI group vs. 0.033 in the comparison group. This difference was greater at Lag9, with standard deviations of 0.249 and 0.152, respectively. See Figure 3 for a visualization of the group and individual performance.

#### *Ad Hoc* Analysis of FSRT Response Time

To ensure that accuracy deficits on the FSRT were not attributable to a speed-accuracy tradeoff given known delayed response times in individuals with TBI (e.g., Incoccia et al., 2004), we completed an *ad hoc* analysis of response time at both lags between participants with and without TBI. We conducted this analysis using an unequal variances *t*-test, as response times are on a continuous distribution. For this analysis, we only included response time for correct trials. Mean response time at Lag1 was 1,550.32 ms (SD: 317.51) for comparison participants and 1,891.89 ms (*SD* = 392.14) for participants with TBI. At Lag9, mean comparison response time was 2,593.12 ms (*SD* = 467.45), and mean response time for participants with TBI was 2,929.89 ms (*SD* = 642.55). Participants with TBI were significantly slower in their overall response time than comparison participants, at both Lag1 (*t*_(76.68)_ = 4.335, *p* < 0.001) and Lag9 (*t*_(71.17)_ = 2.691, *p* = 0.008). We next compared response time for correct items to overall task accuracy, using Spearman’s rho due to the non-continuous nature of the accuracy proportion data. There was a significant negative correlation between overall accuracy and response time for all participants at Lag1 (rho = −0.377, *p* = 0.0005) and Lag9 (rho = −0.293, *p* = 0.008), such that faster response time was associated with improved accuracy at both delays.

### Exploratory Analyses of Relation Between FSRT and Neuropsychological Testing

All 41 participants with TBI and 32 comparison participants completed neuropsychological testing *via* the NIH Toolbox. Comparison participants who did not complete the NIH Toolbox were unable to return to the lab to complete the assessment or had moved away. The participants with TBI performed significantly worse than the comparison participants on both neuropsychological memory assessments. See Table 2 for scores and group differences. Although there was a significant group difference between the performance of participants with TBI and their comparison peers on both neuropsychological assessments, participants with TBI performed well on these standardized assessments of memory. Only three participants with TBI were 1.5 standard deviations below the mean on the Working Memory subtest, and only one participant with TBI was 1.5 standard deviations below the mean on the Episodic Memory Subtest. In contrast, nine participants with TBI were 1.5 standard deviations below comparison mean performance for FSRT Lag1, and 12 participants with TBI were 1.5 standard deviations below the comparison mean at Lag9.

**Table 2:** NIH Toolbox scores for participants with TBI and comparison participants.

	Working memory	Episodic memory
TBI	96.24 (*SD* = 14.87)	102.41 (*SD* = 15.18)
Comparison	114.38 (*SD* = 10.51)	114.78 (*SD* = 17.72)
	*t*_(70.39)_ = 6.096; *p* < 0.001,	*t*_(61.15)_ = 3.15; = *p* = 0.003,
	Cohen’s *d* = 1.409	Cohen’s *d* = 0.750

Given our interest in examining the relative sensitivity of the FSRT to standardized memory measures in detecting relational memory disruptions, we next compared the performance of participants with TBI at FSRT Lag9 to performance on the Episodic Memory subtest using Spearman’s rho. The correlation between proportion correct at Lag9 and the NIH Toolbox Episodic Memory subtest was marginal, rho = 0.304, *p* = 0.053. We next compared performance at FSRT Lag1 to performance on the Working Memory subtest. The correlation between immediate memory for face-scene pairs and performance on the Working Memory subtest was not significant, rho = 0.160, *p* = 0.318.

Per reviewer request, we additionally computed correlations between FSRT accuracy at each lag and both NIH Toolbox subtests for participants with TBI. The additional correlations, which were not part of our planned analysis, are reported in Table 3.

**Table 3:** Correlations (Spearman’s rho) between accuracy at Lag1 and Lag9 on the FSRT and NIH Toolbox working memory and episodic memory subtests.

	NIH working memory	NIH episodic memory
Lag1 accuracy	*rho = 0.159, p = 0.318*	*rho = 0.055, p = 0.735*
Lag9 accuracy	*rho = 0.270, p = 0.087*	*rho = 0.304, p = 0.053*

## Discussion

Although it is well-established that individuals with TBI have memory impairments, we know less about how TBI affects relational memory. We investigated how moderate-severe TBI affects the ability to bind arbitrary relations between elements of an experience, both at short and long lags. Individuals with TBI performed significantly worse than healthy comparison participants, not only after a long lag but also when test trials were presented with no experimenter-imposed delay. An exploratory analysis revealed that performance on the experimental relational memory task did not fully overlap with NIH toolbox assessments of working memory or episodic memory. We discuss each of these findings in more detail below.

Hannula et al. (2006) found that individuals with hippocampal amnesia were impaired relative to demographically matched comparison participants in relational memory binding for face-scene pairings. Although the magnitude of the deficit was larger at long lag (Lag9), it was nonetheless significant as well at short lag (Lag1). The deficit in the Lag1 condition, where there was no experimenter-imposed delay, was striking given the intact performance of individuals with amnesia on working memory tasks such as digit span (Hannula et al., 2006). In the current study with individuals with moderate-severe TBI, we find the same pattern: individuals with TBI perform significantly worse than comparison participants on relational memory binding for face-scene pairings not only at long lags but even at short lags with no experimenter-imposed delay. Interestingly, this discrepancy did not result from a speed-accuracy trade-off, as faster response times were associated with higher accuracy at both delay periods. Our findings show that TBI disrupts relational memory and adds to a growing body of evidence of relational memory deficits in TBI across tasks and severity (Monti et al., 2013; Rigon et al., 2020).

Although there may be some overlap between relational memory and performance on canonical assessments of declarative and working memory, previous work suggests that current standardized neuropsychological assessments may not fully capture, or are not sensitive to, the breadth of relational memory capacities (Rigon et al., 2020). For example, in Hannula et al. (2006), participants with hippocampal amnesia were impaired on the Lag1 condition of the face-scene relation task, despite performing within normal limits on various standardized measures of working memory (e.g., digit span, sentence repetition). Similarly, Rigon et al. (2020) reported that, although performance on a spatial reconstruction task (a measure of spatial relational memory) and performance on the California Verbal Learning Test were correlated, more individuals with TBI were impaired on the spatial reconstruction task than the neuropsychological measure. The authors suggested that the spatial reconstruction task may have increased sensitivity to relational memory deficits (Rigon et al., 2020). Current neuropsychological measures, designed to capture declarative and working memory performance, may not sufficiently tap into relational memory processing across delays.

Here, we found no significant correlation between performance on the FSRT in the short lag (Lag1) condition and the NIH toolbox Working Memory assessment. Indeed, seven participants with TBI who showed a disruption on immediate recall for face-scene pairs performed within the range of normal on the NIH assessment of working memory, and one participant who showed impairment on the NIH assessment was 100% accurate at FSRT Lag1. We also found only a marginal correlation between performance on the FSRT in the long lag (Lag9) condition and the NIH toolbox Episodic Memory assessment, and twelve participants with TBI showed a disruption at FSRT Lag9 but performed within normal limits on the NIH Toolbox Episodic Memory assessment. The participant who exhibited an impairment on the NIH Toolbox Episodic Memory assessment scored 100% at FSRT Lag9 but only responded to three out of eight trials.

More work will allow further exploration of this notion in a larger sample and across a range of relational memory tasks and normative neuropsychological tests of memory. As currently available neuropsychological measures are not designed to specifically capture relational memory performance, our group is actively pursuing this line of work through the development of new experimental tasks and methods that are sensitive to relational memory binding and flexible expression of hippocampal-dependent representations. Our long-term goal is the development of clinically sensitive measures of relational memory that can be easily administered and that are predictive of long-term behavioral outcomes in TBI.

This work is in line with recent calls to infuse cognitive neuroscience advances into the clinical neuropsychology of memory (McAndrews et al., 2020). We used the FSRT here in part because it can detect disruptions in relational memory in a relatively short period with a small number of trials. Bridging the gap between experimental measures and clinical tools will require a thorough investigation of each measure’s psychometric properties, and we plan to further investigate the psychometric properties of the FSRT in future work with larger sample sizes. For example, additional sensitivity analyses using logistic regression will allow for assessment of the relative efficacy of the FSRT to other neuropsychological measures in predicting injury status or functional outcomes. In the meantime, it is worth reflecting on the critical role of memory research in the establishment of the multiple memory systems theory upon which much of neuropsychological assessment is built. As our theories and constructs of memory evolve, so too will the tools we use to measure, characterize, and diagnose memory disorders.

These results provide additional evidence for disruptions in relational memory following moderate-severe TBI and may lead to a better understanding of mechanisms of behavioral dysfunction that affect the everyday lives of individuals with TBI (Morrow et al., 2019). Indeed, impairments in flexible cognition and goal-directed behavior are well-documented in TBI and are often cited as barriers to positive academic, vocational, and interpersonal outcomes (Ylvisaker and Feeney, 1998). Although these deficits are often linked exclusively to frontal lobe abilities, evidence that hippocampal relational memory may be critical in flexible cognition more broadly is intriguing as a potential mechanistic factor (see Rubin et al., 2014). For example, while we focused here on relational memory binding, hippocampal relational memory theory also points to the role of the hippocampus in the flexible expression of relational representations. This flexibility allows for the search, reconstruction, and recombination of elements that make up relational bindings for use in new situations (Konkel and Cohen, 2009). This compositional nature of relational memory permits the retrieval or reactivation of individual, or even new configurations of, bindings established from the rich and complex experiences of our daily lives, allowing the flexible use of relational knowledge across contexts (Eichenbaum and Cohen, 2014).

Given the flexible and adaptive nature of relational memory, it is perhaps unsurprising that disruptions in relational memory, beyond affecting canonical memory functions, have been associated with inflexible cognition and maladaptive behavior across cognitive domains: in communication and language (Duff and Brown-Schmidt, 2012), social cognition (Davidson et al., 2012; Beadle et al., 2013; Spreng, 2013), decision-making (Gupta et al., 2009), perception (Barense et al., 2007; Lee et al., 2012; Aly et al., 2013; Aly and Turke-Brown, 2017), and spatial navigation and environmental exploration (Maguire et al., 2006; Voss et al., 2011a,b; Yee et al., 2014).

We propose that hippocampal-dependent relational memory is critical for flexible and goal-directed behavior, with disruption tied to maladaptive behavior and poor life outcomes. Hippocampal pathology and memory impairments are common in TBI, yet these disruptions have not figured prominently in mechanistic accounts of behavioral dysfunction and long-term outcome beyond the memory deficit itself. The current study, together with other work (Rigon et al., 2020), offers initial tests of this proposal by documenting relational memory deficits in individuals with TBI.

In future studies, we aim to determine if relational memory impairment is predictive of functional outcomes (e.g., employment, social, and community reintegration). For example, of the nine individuals with TBI whose performance was disrupted (off-ceiling) at the short lag in this experiment, five were unemployed or self-employed (e.g., completing odd jobs) at the time of testing, and a sixth became unemployed within 1 week of testing. Although this is a relatively small sample of individuals with TBI, this high proportion of individuals who are unemployed and exhibit a relational memory disruption, even at short delays, is intriguing given the links between relational memory and flexible, goal-directed behavior (Rubin et al., 2014). Further examinations of the role of relational memory in flexible, adaptive behavior promise to have significant implications for understanding the nature of, and potential interventions for, behavioral dysfunction in individuals with TBI.

Although, we hypothesize that relational memory impairment may be linked to hippocampal dysfunction, a limitation of this study is the lack of sufficient neuroimaging or neuroanatomical data to assess the relationship between the structure and function of the hippocampus and FSRT task performance. Future work should move beyond clinical neuroimaging to investigate relationships between task performance and the structure and function of the hippocampus *via* MRI. Relational memory disruptions in individuals with TBI followed a similar pattern to those patients with focal hippocampal damage reported by Hannula and colleagues (i.e., impairment in both lag conditions, including a smaller but still significant deficit at the shortest possible lag), but given the diffuse neural damage that is a hallmark of TBI, it is likely that participants with TBI have damage that extends to other regions outside the hippocampus.

It is interesting in this context that Hannula and Ranganath (2009) found that accuracy on a similar face-scene relational memory task conducted with fMRI was related with increased activation in the lateral prefrontal cortex and functional connectivity between the hippocampus and the prefrontal cortex, whereas hippocampal activity alone predicted relational memory expression *via* eye movement (Hannula and Ranganath, 2009). Because TBI is a disorder of diffuse neural damage and connectivity, ongoing work will be needed to determine the relative contributions and connectivity of the hippocampus and prefrontal cortex in producing relational performance.

Another limitation relates to the generalizability of the results. Our sample exhibited excellent community reintegration, relative to the general population of individuals who sustain a TBI, as measured by employment outcome (Gormley et al., 2019). Of our 41 participants with TBI, twenty-two were gainfully employed outside the home, and five were full-time students. Our sample’s outcomes reflect the heterogeneity of individuals classified as having a moderate-severe TBI by the Mayo Classification Scale, which has led to ongoing conversations in the field about how best to classify and characterize individuals with TBI. Our sample’s average educational attainment of 14.9 years was also above the typically reported average for individuals who sustain a TBI (Gauthier et al., 2018). As such, this sample may not be fully representative of the general population of individuals who sustain a TBI. And, yet, the fact that a sample with such positive functional outcomes exhibited a group relational memory deficit, even at a short delay, is compelling. Future work with larger sample sizes will allow further assessment of the range of, and individual differences in, relational memory performance in this population.

In conclusion, the current study represents a positive step in characterizing relational memory deficits in TBI, regardless of the time domain. We found that individuals with TBI were impaired in relational memory performance, even when testing occurred with no experimenter-imposed delays. Importantly, we demonstrated that relational memory impairments are not fully captured by canonical neuropsychological memory assessments. These results are especially intriguing in characterizing individuals with TBI, given the link between relational memory and flexible, adaptive behavior. Future work characterizing memory impairment in TBI should expand upon these findings, in hopes of identifying sub-groups based on individual differences in relational memory within TBI populations and further examining the psychometric properties of relational memory assessments (Morrow et al., 2019; Rigon et al., 2020). Future investigations of the relationship between relational memory, at both short and long delays, and neuroimaging findings will clarify the nature of this deficit and its link to hippocampal dysfunction. The results of this study indicate that relational memory, even at a short delay, is likely to be impacted by TBI and highlight the need for improved characterization of this deficit, which may have specific consequences for community reintegration and long-term functional outcomes.

## Data Availability Statement

The raw data supporting the conclusions of this article will be made available by the authors, without undue reservation.

## Ethics Statement

The studies involving human participants were reviewed and approved by Human Research Protections Program at Vanderbilt University. The participants provided their written informed consent to participate in this study.

## Author Contributions

EM, MRD, NC, and MCD contributed to the design, analysis, and preparation of the manuscript.

## Conflict of Interest

The authors declare that the research was conducted in the absence of any commercial or financial relationships that could be construed as a potential conflict of interest.

## Footnotes

^1^Per reviewer request, we also conducted these analyses with all trials included (i.e., including trials for which participants did not provide a response and counting them as incorrect). With no-response trials counted as incorrect, mean proportion correct at Lag1 was 0.933 (*SD* = 0.116) for participants with TBI and 0.991 (*SD* = 0.033) for comparison participants. Mean proportion correct at Lag9 was 0.640 (*SD* = 0.252) for participants with TBI and 0.811 (*SD* = 0.151) for comparison participants. Participants with TBI performed significantly worse than comparison participants at Lag1 (*U* = 1,056, *p* = 0.002) and Lag9 (*U* = 1176.5, *p* = 0.0007). When no-response trials were counted as incorrect, 13 participants with TBI had off-ceiling performance at Lag1, relative to three comparison participants.
